# Aquaporin-4 suppresses neuronal pyroptosis after ischemic stroke via the IκBα/NF-κB signaling pathway

**DOI:** 10.3389/fimmu.2026.1778802

**Published:** 2026-03-06

**Authors:** Heling Chu, Jingwei Pan, Qihao Guo, Chuyi Huang

**Affiliations:** 1 Department of Gerontology, Shanghai Sixth People’s Hospital Affiliated to Shanghai Jiao Tong University School of Medicine, Shanghai, China; 2 Health Management Center, Renji Hospital, School of Medicine, Shanghai Jiaotong University, Shanghai, China

**Keywords:** aquaporin-4, cerebral ischemia, neuroinflammation, NF-κB signaling, pyroptosis

## Abstract

**Background:**

Ischemic stroke, a predominant cause of global mortality and disability, involves complex pathophysiological processes where neuroinflammation and pyroptosis play a crucial role. We aimed to investigate the role of the brain’s major water channel Aquaporin-4 (AQP4) in regulating neuronal pyroptosis, a highly inflammatory form of cell death, following cerebral ischemia.

**Methods:**

Utilizing integrated *in vivo* and *in vitro* approaches, we employed AQP4 knockout mice subjected to middle cerebral artery occlusion/reperfusion (MCAO/R) and neuron-astrocyte co-cultures under oxygen-glucose deprivation/reoxygenation (OGD/R) with AQP4 knockdown to investigate the association between AQP4 and neuronal pyroptosis. Also, the downstream pathway was studied via RNA sequencing analysis and the following validation experiment.

**Results:**

Our results demonstrated that AQP4 deficiency significantly worsened neurological deficits, enlarged infarct volume, and intensified oxidative stress. Crucially, AQP4 loss markedly exacerbated neuronal pyroptosis in both the ipsilateral and contralateral cortices *in vivo*, and in cultured neurons *in vitro*. This was evidenced by the specific up-regulation of the NLRP1 inflammasome, increased cleaved caspase-1, and elevated expression of gasdermin D (GSDMD), alongside heightened release of pro-inflammatory cytokines (IL-1β, IL-18, IL-6, TNF-α). RNA sequencing analysis of AQP4-knockdown neurons revealed the nuclear factor-kappa B (NF-κB) signaling pathway as a key downstream target. Mechanistic validation showed that AQP4 deficiency down-regulated NF-κB inhibitor-alpha (IκBα, encoded by *NFKBIA*), leading to increased nuclear translocation and activity of the NF-κB p50/p65 heterodimer. Subsequent gain- and loss-of-function experiments confirmed that NFKBIA/IκBα mediated the anti-pyroptotic effect of AQP4.

**Conclusion:**

Our findings establish AQP4 as a critical suppressor of neuronal pyroptosis after ischemic stroke. It confers protection by enhancing IκBα expression to inhibit NF-κB signaling, thereby dampening NLRP1 inflammasome activation and the subsequent pyroptotic cascade. This study unveils a novel AQP4/IκBα/NF-κB axis in post-ischemic neuroinflammation and highlights AQP4’s role in mitigating remote secondary injury, offering new insights for developing neuroprotective strategies targeting global brain resilience.

## 1 Introduction

Ischemic stroke, which constitutes approximately 65.3% of all cases, is the most prevalent type of stroke (1). Acute ischemic stroke is a major cause of death and disability globally (2). During ischemic stroke, brain tissue experiences a deprivation of blood supply, triggering a cascade of cell death pathways (3). Importantly, as a key pathophysiological process, neuroinflammation both drives the progression of cerebral ischemia and causes subsequent neuronal damage and death (4). Pyroptosis is an inflammatory-dependent programmed cell death that is primarily driven by inflammasome-activated caspases (e.g. caspase-1) and marked by membrane pore formation, cell swelling with eventual plasma membrane rupture, chromatin fragmentation, and the consequent release of pro-inflammatory cellular contents (5, 6). It has been demonstrated that neuronal pyroptosis is crucial for ischemic brain injury (7). However, the mechanism of neuronal pyroptosis after cerebral ischemia remains controversial, and the effective intervention measures is also lacking.

Aquaporins (AQPs) are a group of highly selective membrane channel proteins for water transport with AQP4 being the most predominantly expressed in the brain (8). AQP4 is specifically localized on astrocytes and ependymal cells, especially enriched in the astrocytic end feet facing blood vessels and the pia mater, and is also distributed on brain capillary endothelial cells and the astrocytic processes surrounding synapses (9). Despite of the probability of cytotoxic edema aggravation in in acute cerebral ischemia, AQP4 has also multiple neuroprotective effects (10). For instance, AQP4 participants in blood-brain barrier (BBB) permeability reduction, apoptosis inhibition, neurogenesis promotion, and neuroinflammation alleviation (11, 12). It has been reported that AQP4 is related to pyroptosis in myocardial ischemia (13). However, there has been no research on the relationship between AQP4 and pyroptosis in ischemic stroke.

Ischemic injury is not limited to the infarct focus and its surrounding tissues, functional abnormalities can also occur in areas distant from the infarct lesion, which is referred to as diaschisis (14). The protection and repair of damage at remote areas are of importance for facilitating the recovery of neural function following cerebral ischemia. It has been reported that pyroptosis is not only detected in the peri-infarct area, but also in the contralateral hemispheres after stroke (15). Thus, reducing the contralateral ischemic injury may be also benefit to neurorestoration.

In this work, we aimed to investigate the role of AQP4 on neuronal pyroptosis via both *in vivo* and *in vitro* cerebral ischemic models. Especially, AQP4’s effects were studied at both ipsilateral and contralateral cortices in the *in vivo* experiment. Moreover, we also explored the downstream pathway of AQP4 using RNA sequencing analysis and the following validation experiments.

## 2 Materials and methods

### 2.1 Generation of AQP4 knockout (AQP4^-/-^) mice

We generated AQP4 deficient transgenic knockout mice through targeted gene disruption following a previously established method (16). Briefly, we constructed an AQP4 replacement targeting vector using positive–negative selection cassettes. These cassettes were derived from two vectors: pPolII long neo bpA (containing the *neo^R^* gene) and pXhoMC1TK (containing the *HSVtk* gene). Following linearization with NotI, the targeting construct was electroporated into E14K ES cells. Selection of G418/gancyclovir-resistant ES cell clones was followed by their isolation, amplification, and screening for targeting fidelity through Southern blot analysis. We microinjected cells from two targeted clones into C57BL/6J blastocysts and surgically implanted them into pseudopregnant recipients. The resulting offspring were genotyped via PCR analysis of DNA obtained from tail biopsies. We utilized the following oligonucleotide primers: wild-type (WT) AQP4 forward (5’-ACCATAAACTGGGGTGGCTCAG-3’), WT AQP4 reverse (5’-TAGAGGATGCCGGCTCCAATGA-3’), and Neo (5’-CACCGCTGAATATGCATAAGGCA-3’). We periodically used Southern blot analysis to confirm the fidelity of the polymerase chain reaction (PCR) results. We then generated homozygous AQP4 knockout mice by intercrossing heterozygous mutants. As shown in **Supplementary Figure 1**, WT allele yielded a 240-base pairs (bp) product, heterozygote allele yielded a 240-bp and 320-bp product, and hemozygote allele yielded a 320-bp product.

Male WT and AQP4^-/-^ mice aged 3–4 months were kept in a 12 h/12 h light-dark cycle with free access to food and water under standard laboratory conditions (temperature: 22 ± 2 °C; humidity: 40%). Our animal studies and protocol were approved by Institutional Animal Care and Use Committee of Shanghai Sixth People’s Hospital Affiliated to Shanghai Jiao Tong University School of Medicine. AQP4^-/-^ mice were randomly divided into four groups: sham operation, middle cerebral artery occlusion/reperfusion (MCAO/R) 1 d, MCAO/R 3 d, and MCAO/R 7 d to determine the time point with most severe neural damage and pyroptosis. Then we compared multiple parameters between WT and AQP4^-/-^ mice at the time point.

### 2.2 Mouse MCAO/R model

After anesthetized by isoflurane via a mask (3% for induction, 1.5% for maintenance in 70% nitrous oxide and 30% oxygen), the mice were subjected transient focal ischemia as reported by our previous work (11). The left common carotid, external carotid artery (ECA), and internal carotid artery (ICA) arteries were carefully exposed. Subsequently, a 4–0 monofilament nylon suture with a heat-rounded tip was introduced via the ECA and advanced into the ICA lumen until it occluded the origin of MCA. After one hour of occlusion, reperfusion was initiated by withdrawing the suture completely out of the ECA lumen. Regional cerebral blood flow in the MCA territory was monitored using a laser-Doppler probe (Periflux 5000, Perimed, Sweden) affixed to the skull at a site corresponding to 1 mm posterior and 5 mm lateral to the bregma. Mice were included based on a pre-defined threshold of ≥80% flow reduction during ischemia to verify successful occlusion. Sham-operated controls underwent the same surgical procedure without suture introduction.

### 2.3 Neurological testing

The neurological function after MCAO/R was quantified via Zea-Longa and Garcia scoring systems. The Zea Longa scoring system (a five-point scale), a well-established method for evaluating post-ischemic neurological deficits, is based on observations of posture and motor function. The scoring was conducted as follows: 0) no deficit (normal activity); 1) mild deficit (failure to fully extend the contralateral forepaw); 2) moderate deficit (circling behavior); 3) severe deficit (leftward leaning); 4) critical deficit (no movement and loss of consciousness) (17). Garcia scoring system comprises six items: spontaneous activity, limb movements, forepaw extension symmetry, climbing (on a wire cage), body proprioception, and tactile response. The total score is inversely proportional to neurological function, with a maximum score of 18 indicating a normal, unimpaired mouse and a minimum score of 3 representing severe neurological dysfunction (18).

### 2.4 Measurement of infarct volume

The mice were euthanized by cervical dislocation. After extraction, the brains of mice were sectioned coronally into 1-mm thick slices. The brain slices were then incubated in a 2% solution of 2,3,5-triphenyltetrazolium chloride (TTC, Beyotime Biotech Inc, Suzhou, China) at 37 °C for 30 minutes. Subsequently, the stained slices were fixed in 10% formalin for photography. We determined the infarct area in each slice using image analysis software (Image-Pro Plus, Version 6.0, USA). the percentage of hemispheric infarct volume was calculated according to the formula: infarct volume/whole brain volume×100%.

### 2.5 Western blot

The protein from brain tissues and cells was extracted and Western blot was performed as our previous report (19). The primary antibodies included Anti-AQP4 (1:1000, Abcam, Cambridge, UK), anti-gasdermin D (GSDMD) (1:1000, Abcam), anti-NOD-like receptor protein 1 (NLRP1), (1:1000, Abcam), anti-NLRP2 (1:1000, Sigma-Aldrich, St Louis, MO, USA), anti-NLRP3 (1:1000, Abcam), anti-cleaved caspase-1 (1:1000, Abcam), anti-phosphorylated p38 mitogen-activated protein kinase (p-p38 MAPK) (1:1000, Abcam), anti-total p38 MAPK (t-p38 MAPK) (1:1000, Abcam), anti-p-Akt (1:1000, Sigma-Aldrich), anti-t-Akt (1:1000, Abcam), anti-IκBα (1:1000, Sigma-Aldrich), anti-nuclear factor kappa-B (NF-κB) p50 (1:1000, Sigma-Aldrich), and anti-NF-κB p65 (1:1000, Sigma-Aldrich). Horseradish peroxidase labeled goat anti-rabbit antibody (1:4000, Abcam) was used as secondary antibody. Glyceraldehyde 3-phosphate dehydrogenase (GAPDH) or β-Actin functioned as an internal control.

### 2.6 Hematoxylin and eosin staining

After the mice were euthanized, the brain tissues were collected, fixed in 10% formaldehyde, embedded in paraffin, and sectioned into 5 μm slices. Following staining with hematoxylin and eosin, the dewaxed paraffin sections were sequentially immersed in absolute ethanol, n-butanol, and xylene, and then sealed with neutral gum. Histological images were captured under a microscope.

### 2.7 Biochemical analysis

After the protein was extracted from brain tissues or supernatant, the activity of superoxide dismutase (SOD), glutathione peroxidase (GSH-Px), Catalase (CAT) and levels of methane dicarboxylic aldehyde (MDA) were measured by using commercially available kits (Jiancheng Bioengineering Institute, Nanjing, China), following the manufacturer’s instructions.

### 2.8 Enzyme−linked immunosorbent assay

The concentrations of interleukin-1β (IL-1β), IL-18, IL-6, and tumor necrosis factor-α (TNF-α) were quantified with mouse ELISA kits (R&D Systems Inc., Minneapolis, USA). Brain tissue or supernatant from co-culture homogenates were prepared, and assays were performed according to the manufacturer’s recommendations.

### 2.9 Quantitative real‐time polymerase chain reaction analysis

Total RNA was extracted from the cortical tissue using Trizol Reagent (Sigma-Aldrich). Then, the PrimeScriptTM RT reagent kit (Takara, Shiga, Japan) was used to reverse-transcrib RNA from each sample was to cDNA. Using an ABI PRISM 7500 system (Applied BioSystems, Waltham, USA), the qRT PCR was carried out via a SYBR Green Master Mix (Sigma-Aldrich). The mRNA expression levels were normalized to GAPDH gene using the 2−ΔΔCT method (20). **Table 1** included the sequences of the primers.

**Table 1:** Primer sequences used for qRT-qPCR.

Gene	Forward primer (5’-3’)	Reverse primer (5’-3’)
*NLRP1*	ATACGAAGCCTTTGGGGACT	CACCGCTTCTCTCATCACAA
*NLRP2*	AAGGTCCCCGGATGAACAAC	TCCAGTGCAGAGCTGTTGAG
*NLRP3*	TATCCACTGCCGAGAGGTGA	TCTTGCACACTGGTGGGTTT
*caspase-1*	CTATGGACAAGGCACGGGAC	TCAGCTGATGGAGCTGATTGA
*GSDMD*	AGTGCTCCAGAACCAGAACCG	TCTGCCCTGAATGTTCCCATC
*IL-1β*	AGAGCCCATCCTCTGTGACT	GCTTGGGATCCACACTCTCC
*IL-18*	CCTTTGAGGCATCCAGGACA	GGGAACAGCCAGTGTTCAGT
*IL-6*	CCAGTTGCCTTCTTGGGACT	GTCTCCTCTCCGGACTTGTG
*TNF-α*	AGCCGATGGGTTGTACCTTG	ATAGCAAATCGGCTGACGGT
*NFKBIA*	GCATCGTGGAGCTTTTGGTG	GACATCAGCCCCACACTTCA
*NF-κB1*	ATGGCAGACGATGATCCCTAC	TGTTGACAGTG GTATTTCTGGTG
*RELA*	AGTGTGTGAAGAAGCGAGACC	AAATCGGATGTGAGAGGACAG
*GAPDH*	AGGTCGGTGTGAACGGATTTG	TGTAGACCATGTAGTTGAGGTCA

### 2.10 Immunofluorescence

Mice were anesthetized at 3 d after MCAO, and were perfused with normal saline solution followed by 4% ice-cold paraformaldehyde via the left cardiac ventricle. The brain tissues were then removed and underwent gradient dehydration. Serial coronal sections with a thickness of 10 μm were cut using a freezing microtome, and the sections were blocked with 10% goat serum after washing. The sections were incubated overnight at 4 °C with anti-GSDMD and anti-NeuN polyclonal antibodies (1:500, Abcam). Then, they were incubated for 1 h at 37 °C with tetramethyl rhodamine isothiocyanate (TRITC) or fluorescein isothiocyanate (FITC) coupled secondary antibody (1: 500, Invitrogen). The sections were stained with 4’,6-diamidino-2-phenylindole (DAPI) to visualize the nuclei (12).

For quantitative pyroptotic neurons counting, six non-overlapping fields per coronal section in the ipsilateral or contralateral cortex were randomly selected under high-power magnification. The mean value across these fields was defined as the GSDMD/NeuN count. Field selection was conducted by an investigator blinded to group assignments.

### 2.11 Lentivirus transfection

The transfection of lentivirus to the cells was performed as previous report (21). The short hairpin RNA (shRNA) specific for the mouse *AQP4*, *NLRP1*, and *NFKBIA* genes and the corresponding negative control (NC) shRNA was purchased from a commercial supplier (GenePharma, Suzhou, China). Meanwhile, *NFKBIA* and *NLRP1* genes were subcloned into the vector pcDNA3.1 to generate the vector pcDNA3.1-NFKBIA and the vector pcDNA3.1-NC functioned as control (GenePharma).

### 2.12 Co-culture of primary astrocytes and neurons

We performed co-culture of primary astrocytes and neurons as previous studies (22, 23). Briefly, brains of neonatal mice were bisected along the sagittal plane. After careful removal of the meninges and extraction of most white matter, the cortical tissue was placed in an incubator for digestion with 0.25% trypsin- Ethylene Diamine Tetraacetic Acid (Gibco, Carlsbad, CA, USA). The cortical tissues were then resuspended in medium containing 10% fetal bovine serum (FBS, Gibco). Following filtration through a 70 μm cell strainer, the neuronal and astrocyte suspensions were centrifuged at 900 rpm and 1200 rpm, respectively, for 5 minutes each. The astrocyte precipitates were resuspended in Dulbecco’s Modified Eagle Medium (DMEM)/F12 (Gibco) supplemented with 10% FBS. The neuronal precipitates were resuspended in Neurobasal-A medium containing 10% FBS, 2% B27, 0.25% GlutaMAX, and 1% penicillin/streptomycin (all from Gibco). The neuron-astrocyte co-culture was established using Transwells (Corning, 0.4 μm pores, NY, USA). Astrocytes were plated in the upper compartment, while neurons were plated in the lower compartment. The astrocytes were subcultured at 3-day intervals, and this process was repeated twice. For the subsequent co-culture with mature neurons, astrocytes were first subjected to AQP4 knockdown and then subcultured. The co-culture system was established and maintained for 24 h. The culture medium contained DMEM with 10% FBS and penicillin–streptomycin.

### 2.13 Oxygen-glucose deprivation/reoxygenation

The OGD procedure was conducted by placing the cultures in a 37 °C incubator within an anaerobic chamber, following established protocols (24). The co-cultures were washed with phosphate-buffered saline (PBS) and subsequently incubated in glucose-free Earle’s balanced salt solution (Gibco). Cultures were exposed to an anaerobic environment of 95% N_2_/5% CO_2_, under which the oxygen partial pressure reached 10–15 Torr verified by an oxygen microelectrode at 3 h. Reoxygenation was achieved by returning the cultures to the original medium and placing them in a normoxic chamber.

### 2.14 Cell viability assay

Cell viability was determined via the 3-(4, 5-dimethylthia-zol-2-yl)-2, 5-diphenyltetrazolium bromide (MTT) assay using a MTT Cell Proliferation and Cytotoxicity Assay Kit (Beyotime). After the neurons were added to a 96-well plate, 10 μL of an MTT solution (5 mg/mL) were added to the wells and the plate was incubated for an additional 4 h. Absorbance was measured at 570 nm using a microplate reader (Olympus, Tokyo, Japan). The percentage of viable cells was then calculated by comparing the readings to those from untreated cells.

### 2.15 Lactate dehydrogenase release assay

The injury of neurons was quantified using an LDH Cytotoxicity Assay Kit (Beyotime) according to the manufacturer’s instructions. Absorbance was measured at 490 nm using a microplate reader. The cytotoxicity was calculated using the formula: Cytotoxicity (%) = (A490 Test Sample - A490 Low control)/(A490 High Control -A490 Low Control) × 100%.

### 2.16 RNA sequencing analysis

Total RNA was isolated from control and AQP4 knockdown neurons using TRIzol™ reagent (Thermo Fisher Scientific). RNA integrity was assessed using an Agilent 2100 Bioanalyzer (Agilent Technologies), with all samples exhibiting an RNA Integrity Number (RIN)>8.0. RNA concentration was measured using a NanoDrop 2000 spectrophotometer (Thermo Fisher Scientific). Strand-specific cDNA libraries were prepared using the Illumina TruSeq Stranded mRNA LT Sample Prep Kit (Illumina) according to the manufacturer’s protocol. Briefly, poly-A containing mRNA was purified, fragmented, and reverse-transcribed into cDNA. Following end repair, A-tailing, and adapter ligation, libraries were amplified by PCR. Library quality and size distribution were verified using an Agilent 2100 Bioanalyzer. Qualified libraries were sequenced on an Illumina NovaSeq 6000 platform to generate 150-bp paired-end reads, with a minimum sequencing depth of 40 million raw reads per sample. Raw sequencing reads were quality-filtered using fastp to remove low-quality bases, adapter sequences, and short reads. Clean reads were then aligned to the reference genome (GRCm38) using HISAT2 (v2.2.1). The average alignment rate exceeded 90% for all samples. Gene-level read counts were obtained using feature Counts (v2.0.1). Expression levels were normalized using Transcripts Per Million (TPM). Differential expression analysis was performed using the DESeq2 package (v1.38.3) in R. Genes with an absolute log2 fold change ≥1 and false discovery rate (FDR)-adjusted *P* value< 0.05 were considered significantly differentially expressed genes (DEGs). Gene Ontology (GO) and Kyoto Encyclopedia of Genes and Genomes (KEGG) pathway enrichment analyses were performed on the list of DEGs using the cluster Profiler R package (v4.6.2). Terms with FDR-adjusted *P* value<0.05 were considered significantly enriched.

### 2.17 Statistical analysis

All data were presented as mean ± standard deviations after a normal distribution test was performed. Statistical analysis was carried out using SPSS22.0. Depending on the number of groups, either a two-tailed Student *t* test or one-way analysis of variance (ANOVA) was employed for statistical comparisons. When ANOVA indicated significant differences, Tukey’s honestly significant difference test was used for intergroup comparisons. The statistical significance level was defined as *P* < 0.05. GraphPad Prism 9.00 software was utilized for figure creation.

## 3 Results

### 3.1 The comparison of neurological function and pyroptosis at different time points in AQP4^-/-^ mice

**Figure 1:**
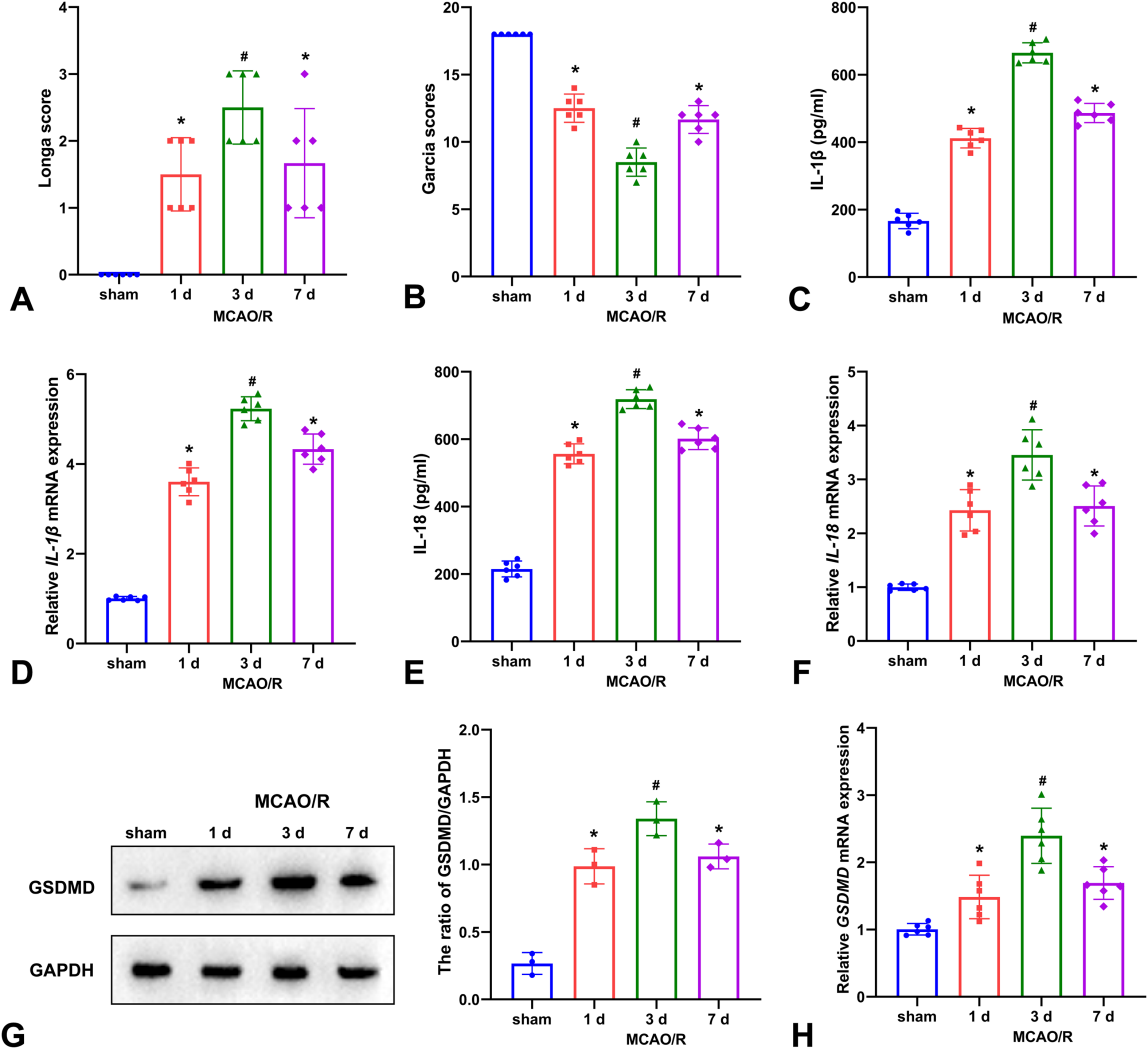
The comparison of neurological function and pyroptosis at different time points in AQP4^-/-^ mice. The Zea Longa scores **(A)** were highest and the Garcia scores **(B)** were lowest at 3 d after MCAO/R compared with 1 d and 7 d in AQP4^-/-^ mice. The expression of IL-1β **(C)**, *IL-1β* mRNA **(D)**, IL-18 **(E)**, and *IL-18* mRNA **(F)** assessed by ELISA and qRT-PCR was also increased at 3 d after MCAO/R compared with other time points. Representative western blot images and hemi-quantitative analysis of GSDMD protein **(G)** and *GSDMD* mRNA **(H)** expression peaked at 3 d after MCAO/R. *P < 0.05 *vs*. sham; #*P* < 0.05 *vs*. other groups.

The highest Zea Longa scores and lowest Garcia scores were observed at 3 d after MCAO/R in AQP4^-/-^ mice, indicating that the worst neurological function was at this time point (**Figures 1A, B**). Similarly, the proteins tested by ELISA and mRNAs tested by qRT-PCR of IL-1β and IL-18 were increased at 3 d after MCAO/R compared with other time points (**Figures 1C-F**). Importantly, both Western blot and qRT-PCR demonstrated the peak of the key pyroptotic protein GSDMD was also detected at 3 d after MCAO/R (**Figures 1G, H**). The results indicated that the most significant pyroptosis in AQP4^-/-^ mice was observed at 3 d after MCAO/R. Thus, we selected this time point for the following experiments.

### 3.2 The influence of AQP4 knockout on infarct volume, neurological function, neuron numbers, and oxidative stress

**Figure 2:**
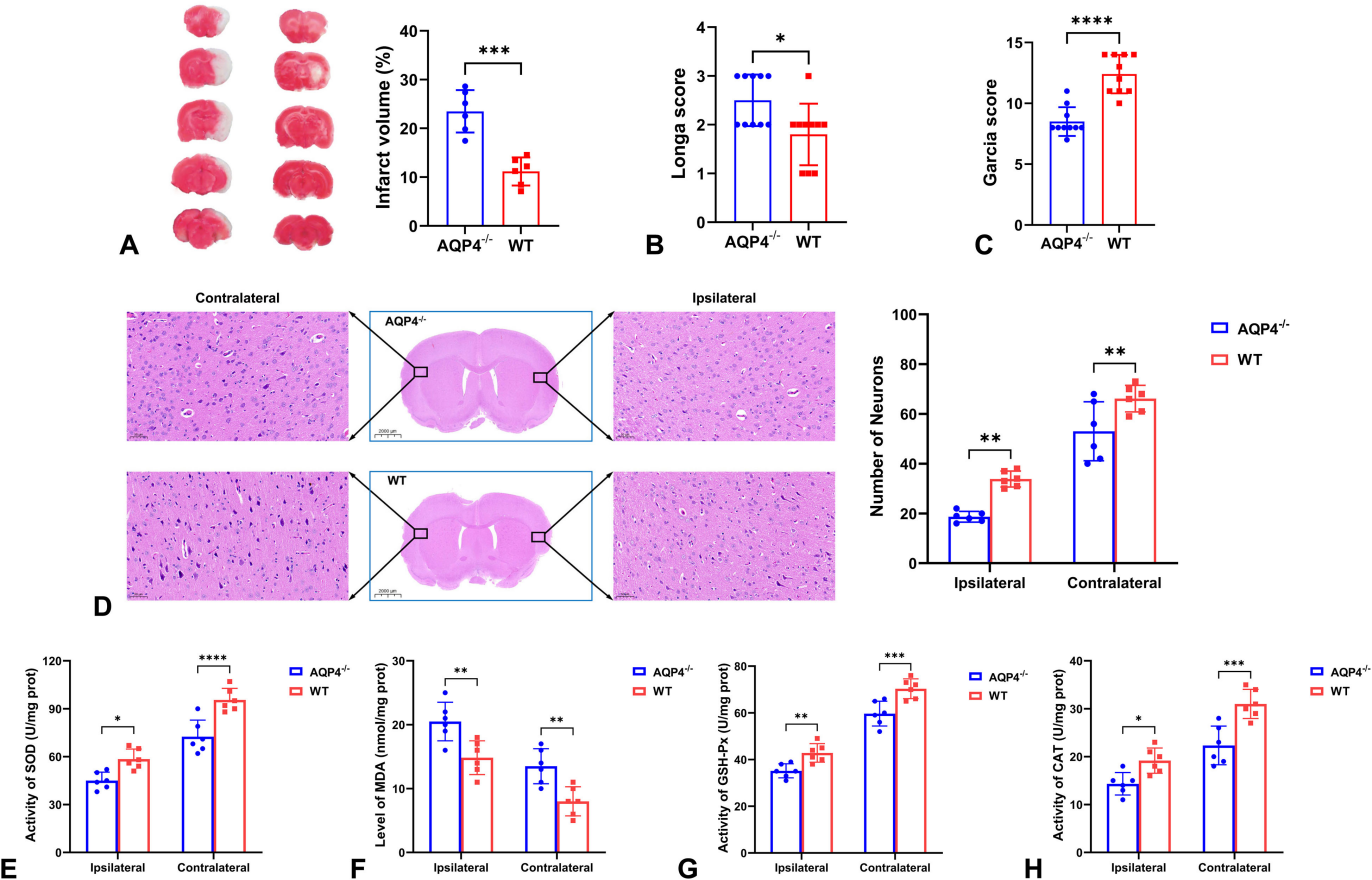
The influence of AQP4 knockout on infarct volume, neurological function, neuron numbers, and oxidative stress. The larger infarct volume **(A)** and worse neurological function assessed by Longa score **(B)** and Garcia score **(C)** were observed in AQP4^-/-^ mice compared with WT mice at 3 d after MCAO/R. Higher numbers of neurons assessed by HE staining in both ipsilateral and contralateral cortices were in WT mice than AQP4^-/-^ mice **(D)**. AQP4 knockout led to more severity of oxidative stress at bilateral cortices evaluated by lower activity of SOD **(E)**, GSH-Px **(G)**, and CAT **(H)** as well as higher level of MDA **(F)**. *P < 0.05; **P < 0.01; ***P < 0.001; ****P < 0.0001.

TTC staining showed that AQP4^-/-^ mice had larger infarct volume compared with WT mice at 3 d after MCAO/R (**Figure 2A**). Moreover, both Zea-Longa and Garcia scoring systems revealed worse neurological function in AQP4^-/-^ mice compared with WT mice (**Figures 2B, C**). Meanwhile, there were more neurons assessed by HE staining in both ipsilateral and contralateral cortices in WT mice than AQP4^-/-^ mice (**Figure 2D**). AQP4 knockout led to more severity of oxidative stress evaluated by lower activity of SOD, GSH-Px, and CAT as well as higher level of MDA (**Figures 2E-H**).

### 3.3 The comparison of neuronal pyroptosis between AQP4^-/-^ and WT mice in the ipsilateral and contralateral cortices

**Figure 3:**
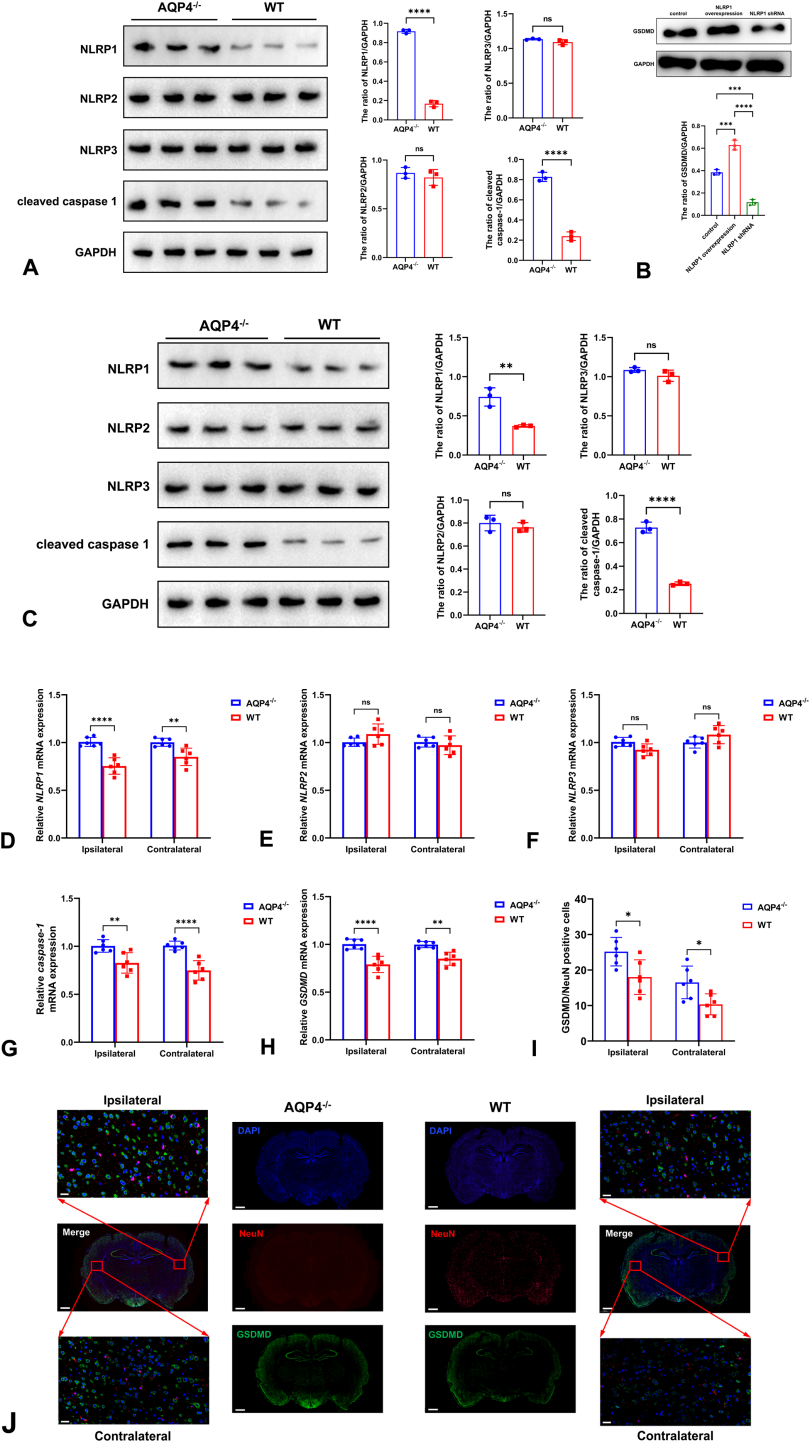
Neuronal pyroptosis in AQP4^-/-^ and WT mice. The expression of NLRP1 and cleaved caspase-1 rather than NLRP2 and NLRP3 was significantly increased in AQP4^-/-^ mice than WT mice in the ipsilateral cortex **(A)**. NLRP1 overexpression increased GSDMD expression, while NLRP1 knockdown decreased the expression of GSDMD **(B)**. Similar changing trend of NLRP1, NLRP2, NLRP3, and cleaved caspase-1 was also observed in the contralateral cortex **(C)**. The mRNA expression of *NLRP1***(D)**, *NLRP2***(E)**, *NLRP3***(F)**, *caspase-1***(G)**, and *GSDMD***(H)** tested by qRT-PCR was consistent with their proteins at bilateral cortices. The immunofluorescence images **(I)** and quantitative analysis **(J)** showed that more GSDMD/NeuN positive cells were found in AQP4^-/-^ mice at the bilateral cortices. Scale bar: 2000 μm (low magnification); 40 μm (high magnification). ns *P*>0.05; *P < 0.05; **P < 0.01; ****P < 0.0001.

In the ipsilateral cortex to the infarction, the expression of NLRP1 and cleaved caspase-1 was significantly increased in AQP4^-/-^ mice than WT mice, while no differences of NLRP2 and NLRP3 were detected between the two groups (**Figure 3A**). To further verify whether NLRP1 promoted pyroptosis, we knocked down and overexpressed NLRP1 and observed the change of GSDMD. The result revealed that NLRP1 overexpression increased GSDMD expression, while NLRP1 shRNA decreased the expression of GSDMD (**Figure 3B**). Similar changing trend of NLRP1, NLRP2, NLRP3, and cleaved caspase-1 was also observed in the contralateral cortex (**Figure 3C**). Expectably, the results of qRT-PCR were consistent with Western blot that the mRNAs of *NLRP1*, *caspase-1*, and *GSDMD* rather than *NLRP2* and *NLRP3* were up-regulated in both ipsilateral and contralateral cortices (**Figures 3D-H**). In order to test neuronal pyroptosis, we co-labeled GSDMD and NeuN. Our findings reveled that more GSDMD/NeuN positive cells were found in AQP4^-/-^ mice at the bilateral cortices (**Figures 3I, J**).

### 3.4 The expression of inflammatory cytokines and signal pathways in AQP4^-/-^ and WT mice in the ipsilateral and contralateral cortices

**Figure 4:**
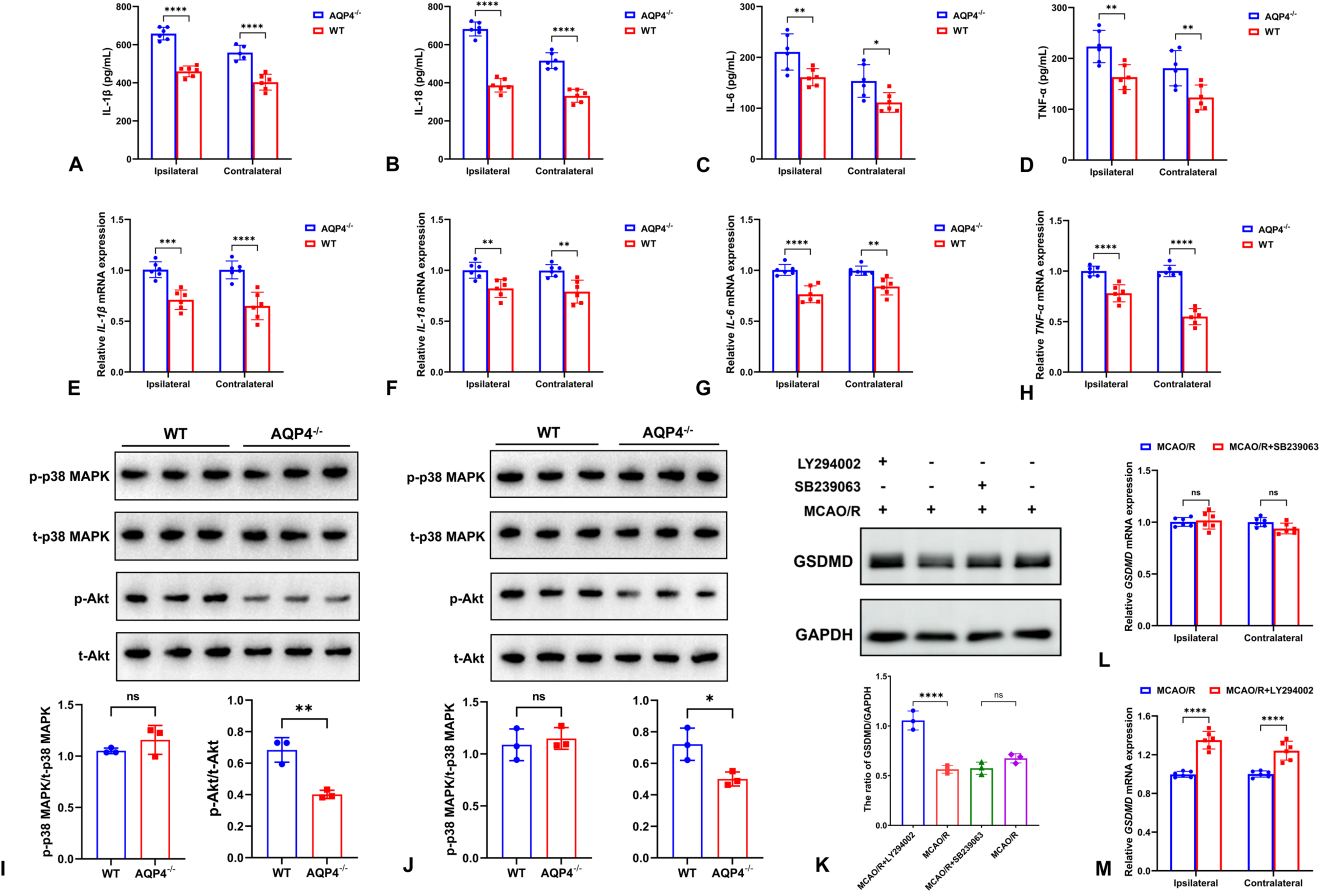
The expression of inflammatory cytokines and signal pathways in AQP4^-/-^ and WT mice. The levels of inflammatory cytokines including IL-1β **(A)**, IL-18 **(B)**, IL-6 **(C)**, and TNF-α **(D)** proteins by ELISA as well as the expression of *IL-1β***(E)**, *IL-18***(F)**, *IL-6***(G)**, and *TNF-α***(H)** mRNAs by qRT-PCR were all higher in AQP4^-/-^ mice than WT mice at both ipsilateral and contralateral cortices. The expression of p-Akt/t-Akt was increased in AQP4^-/-^ mice compared with WT mice at bilateral cortices, while no difference in p-p38 MAPK/t-p38 MAPK between the two groups at ipsilateral **(I)** and contralateral **(J)** cortices. PI3K inhibitor LY294002 reduced GSDMD protein expression in AQP4^-/-^ mice, while p38 MAPK inhibitor SB239063 had no influence in the expression of GSDMD **(K)**. The regulation of *GSDMD* mRNA expression by SB239063 **(L)** and LY294002 **(M)** at bilateral cortices was similar to their proteins. ns *P*>0.05; *P < 0.05; **P < 0.01; ***P < 0.001; ****P < 0.0001.

Western blot and qRT-PCR showed that the levels of inflammatory cytokines including IL-1β, IL-18, IL-6, and TNF-α were all higher in AQP4^-/-^ mice than WT mice at both ipsilateral and contralateral cortices (**Figures 4A-H**). In addition, the expression of p-Akt/t-Akt was increased in AQP4^-/-^ mice compared with WT mice at bilateral cortices, while no difference in p-p38 MAPK/t-p38 MAPK between the two groups (**Figures 4I, J**). Moreover, PI3K inhibitor LY294002 reduced GSDMD expression including protein and mRNA in AQP4^-/-^ mice. However, p38 MAPK inhibitor SB239063 hardly impacted the expression of GSDMD (**Figures 4K-M**). These findings indicated that PI3K/Akt pathway may play a role in the effects of AQP4 on neuronal pyroptosis.

### 3.5 The effects of AQP4 knockdown on neuronal viability, pyroptosis, and oxidative stress in OGD/R model

**Figure 5:**
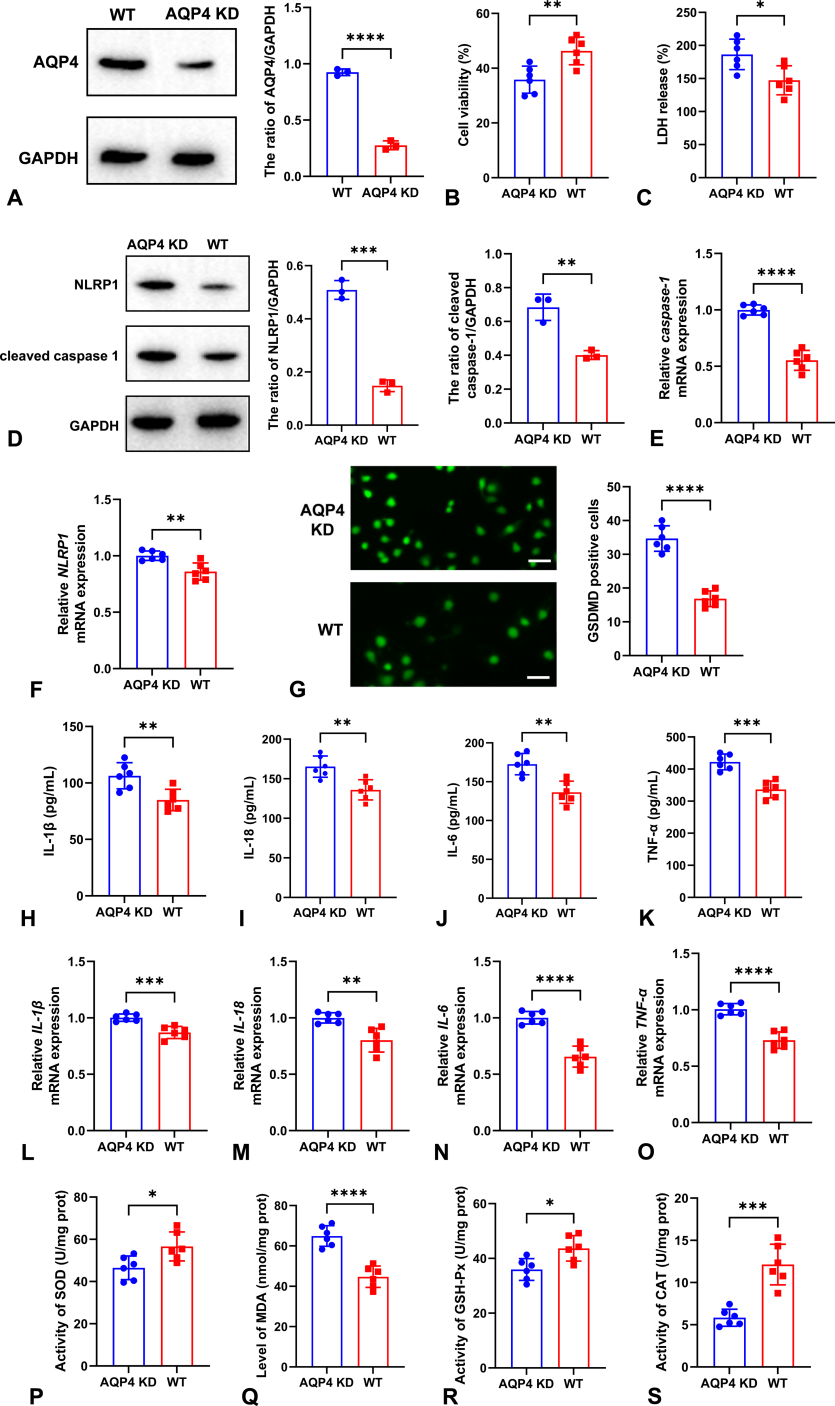
The effects of AQP4 knockdown on neuronal viability, pyroptosis, and oxidative stress in OGD/R model. The expression of AQP4 protein was significantly decreased in AQP4 knockdown groups **(A)**. AQP4 knockdown reduced neuronal viability evaluated by MTT assay **(B)** and increased neuronal cytotoxicity evaluated by LDH release **(C)**. Western blot analysis for the expression of NLRP1 and cleaved caspase-1 proteins **(D)** and qRT-PCR for testing the mRNA expression of *caspase-1***(E)** and *NLRP1***(F)** were all increased in AQP4 knockdown group after OGD/R. The immunofluorescence images and quantitative analysis revealed that neuronal GSDMD expression was markedly elevated after AQP4 was knocked down. Scale bar: 50 μm **(G)**. The levels of inflammatory cytokines including IL-1β **(H)**, IL-18 **(I)**, IL-6 **(J)**, and TNF-α **(K)** as well as their mRNAs including *IL-1β***(L)**, *IL-18***(M)**, *IL-6***(N)**, and *TNF-α***(O)** were higher in AQP4 knockdown group after OGD/R than those in WT group. More severity of oxidative stress evaluated by lower activity of SOD **(P)**, GSH-Px **(R)**, and CAT **(S)** as well as higher level of MDA **(Q)** was detected in AQP4 knockdown group. *P < 0.05; **P < 0.01; ***P < 0.001; ****P < 0.0001.

The expression of AQP4 was significantly decreased after AQP4 knockdown by shRNA (**Figure 5A**). AQP4 knockdown reduced neuronal viability evaluated by MTT assay and increased neuronal cytotoxicity evaluated by LDH release (**Figures 5B, C**). Importantly, the crucial pyroptosis-associated proteins containing NLRP1, cleaved caspase-1, and GSDMD were all increased in AQP4 knockdown group after OGD/R (**Figures 5D-G**). Also, higher levels of inflammatory cytokines such as IL-1β, IL-18, IL-6, and TNF-α (both proteins and mRNAs) were observed in AQP4 knockdown group (**Figures 5H-O**). Also, more severity of oxidative stress evaluated by lower activity of SOD, GSH-Px, and CAT as well as higher level of MDA was detected in AQP4 knockdown group compared with WT group after OGD/R (**Figures 5P-S**).

### 3.6 DEGs and GO and KEGG pathway enrichment analyses in RNA sequencing analysis and further validation

**Figure 6:**
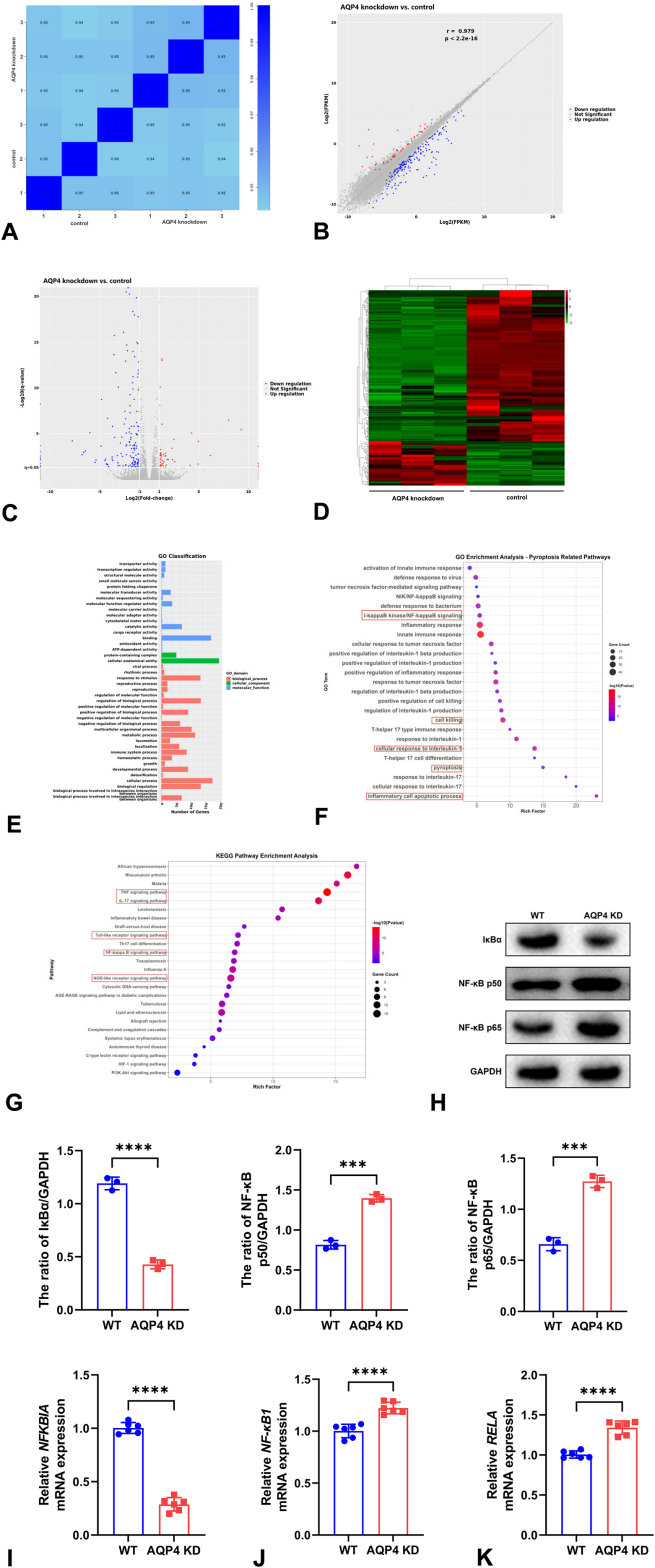
RNA sequencing analysis between WT and AQP4 knockdown groups and further validation. Expression correlation scatter plot showed the Pearson correlation coefficients were above 0.9, indicating good reproducibility among samples within each group **(A)**. The scatter plot revealed a strong positive linear relationship between the two groups, confirming excellent technical reproducibility **(B)**. AQP4 knockdown caused significant up-regulation of 54 genes and down-regulation of 183 genes in neuronal OGD/R models displayed by the volcano plot **(C)** and heatmap **(D)** of DEGs. In the GO analysis, the most represented terms of biological process included cellular process, metabolic process, biological regulation, and response to stimulus; cellular component featured cellular anatomical entity and protein-containing complex; molecular function mainly contained binding, catalytic activity, and transporter activity **(E)**. The bubble plots of top 25 of GO enrichment revealed that the terms most related to pyroptosis included I-κB kinase/NF-κB signaling, cell killing, cellular response to interleukin-1pyroptosis, and inflammatory cell apoptotic process **(F)**. The key pyroptosis-related pathways in KEGG pathway enrichment analysis encompassed TNF signaling pathway, IL-17 signaling pathway, Toll-like receptor signaling pathway, NF-κB signaling, and NOD-like receptor signaling pathway **(G)**. Western blot showed IκBα was reduced while NF-κB p50 and p65 were increased after the knockdown of AQP4 **(H)**. The mRNAs encoding these proteins including *Nfkbia***(I)**, *NF-κB1***(J)**, and *RELA***(K)** were also up-regulated by AQP4 knockdown. ***P < 0.001; ****P < 0.0001.

To identify AQP4-regulated mRNAs in neuronal OGD/R models, we utilized RNA sequencing for analysis, which was performed for comparison between control and AQP4 knockdown groups, with three samples per group. Based on the gene expression results of each sample, we assessed the reproducibility among samples by calculating the Pearson correlation coefficients, which were above 0.9, indicating good reproducibility among samples within each group (**Figure 6A**). Similarly, the scatter plot revealed a strong positive linear relationship between the two groups, as indicated by the Pearson correlation coefficient of 0.979, confirming excellent technical reproducibility (**Figure 6B**). AQP4 knockdown led to significant up-regulation of 54 genes and down-regulation of 183 genes in neuronal OGD/R models displayed by the volcano plot and heatmap of DEGs (**Figures 6C, D**). In the GO analysis, the most represented terms of biological process (BP) included cellular process, metabolic process, biological regulation, and response to stimulus; cellular component (CC) featured cellular anatomical entity and protein-containing complex; molecular function (MF) mainly contained binding, catalytic activity, and transporter activity (**Figure 6E**). Moreover, the bubble plots of top 25 of GO enrichment showed that the terms most related to pyroptosis included I-κB kinase/NF-κB signaling, cell killing, cellular response to interleukin-1pyroptosis, and inflammatory cell apoptotic process (**Figure 6F**). In addition, through KEGG pathway enrichment analysis, the DEGs were categorized into distinct functional pathways. The key pyroptosis-related pathways encompassed TNF signaling pathway, IL-17 signaling pathway, Toll-like receptor signaling pathway, NF-κB signaling, and NOD-like receptor signaling pathway (**Figure 6G**). As both GO and KEGG enrichment analyses revealed NF-κB signaling, it can be regarded as the downstream pathway of AQP4. Importantly, the genes in the DEGs associated with NF-κB signaling was nuclear factor-kappa-B inhibitor-alpha (*Nfkbia*), *NF-κB1*, and *RELA* with FDR of 3.27E-5, 6.54E-4, and 8.22E-6, respectively. Furthermore, we demonstrated both *Nfkbia* and its encoded protein IκBα were decreased in AQP4 knockdown group using qRT-PCR and Western blot (**Figures 6H, I**). Inversely, *NF-κB1* and *RELA genes* as well as the encoded proteins NF-κB p50 and p65 were increased after the knockdown of AQP4 (**Figures 6H, J, K**).

### 3.7 The changes of neuronal pyroptosis and NF-κB signaling after Nfkbia knockdown and overexpression

**Figure 7:**
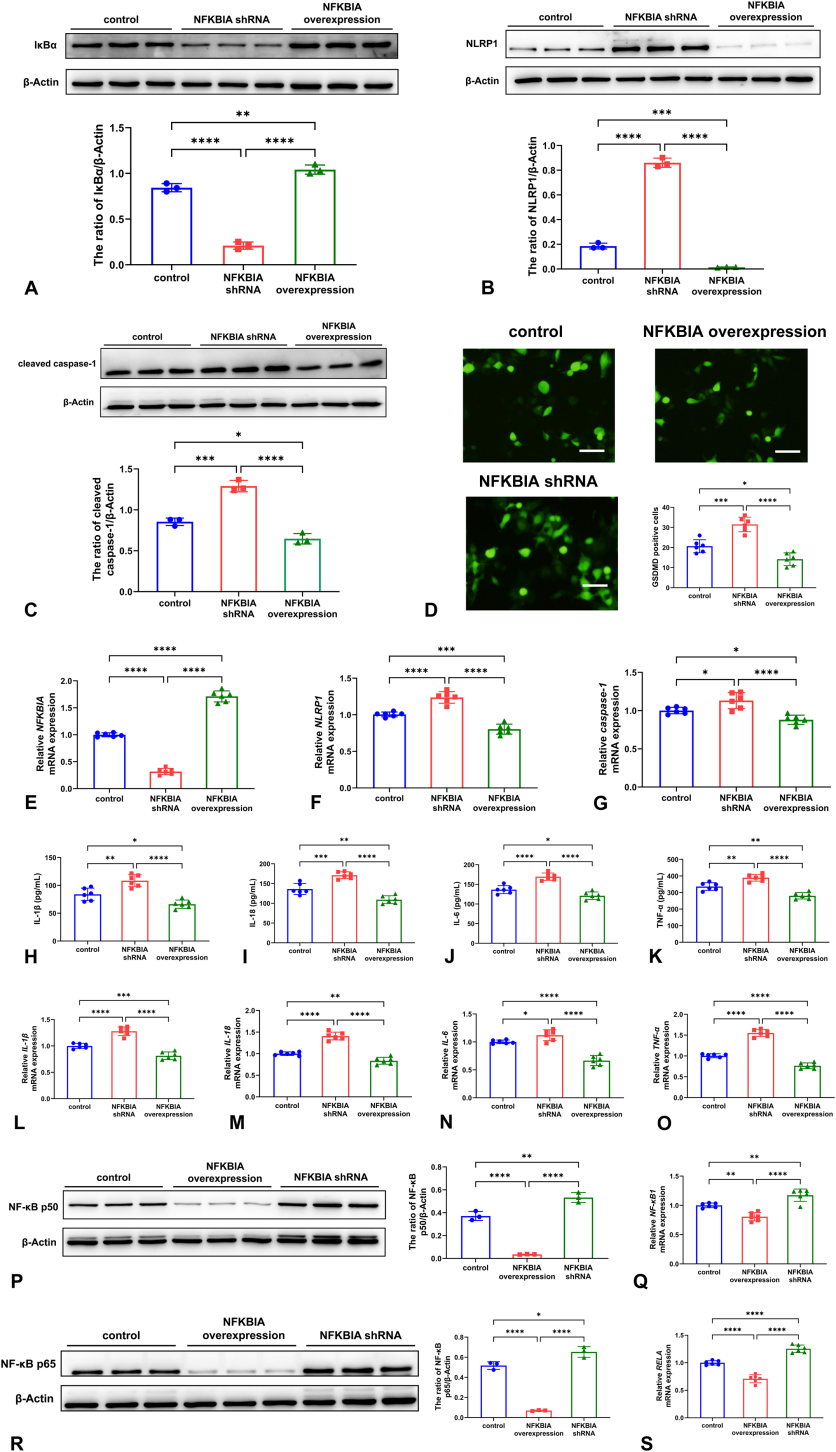
The changes of neuronal pyroptosis and NF-κB signaling after *Nfkbia* knockdown and overexpression. The expression of IκBα was significantly down-regulated after *Nfkbia* knockdown and up-regulated after the overexpression **(A)**. Western blot analysis showed NLRP1 **(B)** and cleaved caspase-1 **(C)** proteins were increased by the knockdown of *NFKBIA*, while were reduced by *Nfkbia* overexpression. Similar changing trend of neuronal GSDMD expression was observed in immunofluorescence images and quantitative analysis of. Scale bar: 50 μm **(D)**. The mRNA expression of *Nfkbia***(E)**, *NF-κB1***(F)**, and *RELA***(G)** after *Nfkbia* knockdown and overexpression was similar to their encoded proteins. Higher levels of inflammatory cytokines such as IL-1β **(H)**, IL-18 **(I)**, IL-6 **(J)**, and TNF-α **(K)** as well as the mRNAs *IL-1β***(L)**, *IL-18***(M)**, *IL-6***(N)**, and *TNF-α***(O)** were observed in *Nfkbia* knockdown group, and *Nfkbia* verexpression group had lower levels. *Nfkbia* knockdown reduced the NF-κB p50 **(P)**, *NF-κB1* mRNA **(Q)**, NF-κB p65 **(R)** and *RELA* mRNA **(S)**, which were inversely elevated after the *Nfkbia* was overexpressed. *P < 0.05; **P < 0.01; ***P < 0.001; ****P < 0.0001.

We knocked down and overexpressed *Nfkbia* via lentivirus transfection in WT mice derived neuron OGD/R model. IκBα was significantly down-regulated after *Nfkbia* knockdown and up-regulated after the overexpression (**Figure 7A**). The crucial pyroptosis-related proteins including NLRP1, cleaved caspase-1, and GSDMD including the proteins and mRNAs were all increased by the knockdown of *Nfkbia*, while were reduced by *Nfkbia* overexpression (**Figures 7B–G**). Moreover, higher levels of inflammatory cytokines such as IL-1β, IL-18, IL-6, and TNF-α were observed in *Nfkbia* knockdown group, and *Nfkbia* overexpression group had lower levels (**Figures 7H–O**). In addition, *Nfkbia* knockdown reduced the NF-κB p50 and p65, which were inversely elevated after *Nfkbia* was overexpressed (**Figures 7P–S**).

## 4 Discussion

In the present study, we provided converging *in vivo* and *in vitro* evidence that AQP4 acts as a critical suppressor of neuronal pyroptosis following ischemic stroke, with its deficiency markedly exacerbating neurological impairment, infarct progression, oxidative stress, and inflammatory responses. Importantly, our work expanded current understanding by demonstrating that AQP4 regulated pyroptosis not only in the ischemic ipsilateral hemisphere but also in the contralateral cortex, highlighting its relevance to remote secondary injury and diaschisis. Using RNA sequencing and mechanistic validation, we further identified NFKBIA/IκBα-dependent NF-κB signaling as a previously unrecognized downstream pathway through which AQP4 modulated neuronal pyroptosis.

Previous studies have described both detrimental and protective roles of AQP4 in ischemic stroke. While AQP4 facilitates water influx contributing to cytotoxic edema (25), it simultaneously supports neurovascular homeostasis, BBB stabilization, metabolic exchange, and neuroinflammatory resolution (26). In addition, the normal structure and function of AQP4 is crucial for alleviating the impairment of glymphatic system after ischemic stroke (27, 28). In line with the latter protective functions, our findings demonstrated that AQP4 knockout led to larger infarct volumes, more profound neurological deficits, and more severe neuronal loss. These results support the notion that the absence of AQP4 impairs key compensatory mechanisms required to preserve neurovascular integrity under ischemic stress, which is in accord with previous reports (10).

A major conceptual advancement of this work is the observation that AQP4 deficiency intensifies pyroptosis in both hemispheres. Earlier evidence has shown that neuronal pyroptosis occurs predominantly in peri-infarct tissue, but evidence for contralateral involvement has been limited (15). Our findings that NLRP1, cleaved caspase-1, and GSDMD were up-regulated bilaterally in AQP4^-/-^ mice provide compelling support that AQP4 contributes to global brain resilience after focal ischemia. This aligns with emerging evidence that contralateral neuronal dysfunction and inflammation are critical components of diaschisis, influencing long-term functional recovery (29). Diaschisis is the dysfunction of brain regions remote from but connected to the primary infarct site, contributing significantly to persistent cognitive and motor disability after stroke, which yet remains insufficiently targeted by current therapies (30). Our data provide the first evidence that neuronal pyroptosis may be a key cellular and molecular mediator of this remote dysfunction. It suggests that attenuating the exacerbated remote pyroptosis associated with AQP4 deficiency could mitigate diaschisis, thereby potentially improving sensory-motor integration, cognitive function, and the overall capacity for neural network compensation and reorganization during recovery. Moreover, interventions targeting AQP4 or its downstream signaling may offer a “dual-target” strategy for simultaneous protection of the ischemic penumbra and vulnerable remote neural networks, promoting global brain resilience. The pathological progression in remote regions may differ from that in the core infarct, potentially unfolding over a more protracted time course. This implies that interventions targeting such remote secondary damage might have a longer therapeutic window, offering novel opportunities for treatment in the subacute and recovery phases.

One of the key mechanistic findings in our study is the selective activation of the NLRP1 inflammasome, with no significant changes in NLRP2 or NLRP3 inflammasome. Although NLRP3 has been widely studied in ischemic neuroinflammation (31), our data demonstrate that NLRP1 may represent a principal pyroptotic driver in neurons lacking AQP4. Previous studies have also reported that NLRP1 rather than other inflammasome plays the key role in pyroptosis (32, 33). It is well-established that NLRP1 is highly and constitutively expressed in neurons, particularly in the cerebral cortex, while NLRP3 is predominantly expressed and activated in microglia and infiltrating macrophages (34, 35). Since our study specifically focuses on neuronal pyroptosis, the observed dominant activation of NLRP1 aligns with the cellular context of our model. In addition, our data consistently show that AQP4 deficiency leads to significantly aggravated oxidative stress in both hemispheres *in vivo* and in neurons *in vitro*, as evidenced by increased MDA levels and suppressed antioxidant enzyme activities. Increased oxidative stress in AQP4^-/-^ mice and AQP4 shRNA treated neurons further supports the view that AQP4 is integral to redox homeostasis, in accord with previous study (36). Oxidative stress is a well-established enhancer of inflammasome activation, and a large body of literature indicates that oxidative stress are potent and preferential activators of the NLRP1 inflammasome (32, 37). Considering interaction may exist between oxidative stress and NLRP1, the loss of AQP4 activates NLRP1 directly or through promoting oxidative stress to induce neuronal pyroptosis via caspase-1 and GSDMD in both hemispheres. Importantly, pyroptosis is a form of regulated cell death characterized by cell lysis and pro-inflammatory signaling (38). Also, our previous studies have revealed that AQP4 plays a role in inhibiting inflammatory cytokines (12, 39). Concurrently, elevated IL-1β, IL-18, IL-6, and TNF-α levels in both *in vivo* and *in vitro* ischemic models demonstrate that AQP4 loss facilitates a robust inflammatory cascade, which further drives secondary injury and bilateral neuronal dysfunction, including pyroptosis. Together, these results position AQP4 as a multifaceted regulator of oxidative homeostasis and neuroinflammation.

We further investigated the downstream pathways of AQP4 to regulate pyroptosis. We first tested the change of two common pathways: MAPK and PI3K/Akt, and found AQP4 knockout increased the expression of p-Akt/t-Akt rather than p-p38 MAPK/t-p38 MAPK. Moreover, PI3K inhibitor suppressed pyroptosis induced by AQP4 loss, indicating AQP4 may alleviate ischemic pyroptosis by activating PI3K/Akt pathway. The PI3K/Akt signaling pathway plays a critical role in mitigating cerebral ischemia-reperfusion injury by alleviating BBB disruption, reducing oxidative stress and endoplasmic reticulum stress, suppressing inflammation, modulating programmed cell death, and ultimately improving pathological outcomes (40). It has been reported that the activation of PI3K/Akt pathway suppresses pyroptosis in cerebral ischemic models (41), which is in accord with our results.

A major strength of this study lies in integrating transcriptomic profiling with functional validation. GO and KEGG enrichment analyses in RNA sequencing indicated NF-κB signaling pathway was the main pathway through which AQP4 regulated pyroptosis. Importantly, the DGEs included *NFKBIA*, *NF-κB1*, and *RELA* genes, all of which are crucial components of NF-κB signaling pathway. We further validated by qRT-PCR and Western blot that the genes and their encoded proteins were all changed after AQP4 knockdown. The NF-κB family is composed of five structurally similar members: RELA (p65), RELB, c-REL, NF-κB1 (p50/p105), and NF-κB2 (p52/p100), which can assemble into different dimeric combinations (42). It is a transcription factor crucial for a wide array of cellular processes, coordinating immune responses, cell survival, and development via intricate activation pathways and interactions with diverse cellular components (43). It has been reported that inhibiting NF-κB pathway can suppress pyroptosis and neuroinflammation after ischemic stroke (44, 45). Moreover, AQP4 overexpression suppresses phosphorylation of NF-κB pathway, consequently reducing the amplification of neuroinflammation and mitigating neuronal injury (46). Therefore, according to our findings, the presence of AQP4 inhibits NF-κB signaling pathway by increasing IκBα expression and reducing the NF-κB complex composed by p50/p65 heterodimer, and then alleviates neuroinflammation and neuronal pyroptosis.

Indeed, AQP4 is primarily expressed in astrocytes, and its modulation likely influences neuronal pyroptosis through astrocyte-neuron interactions. We have demonstrated in our study that AQP4 knockdown elevates inflammatory cytokines in the supernatant from astrocyte-neuron co-culture, which is in accord with our previous studies (12, 39). It indicates that astrocytes with impaired AQP4 function may alter the secretion of cytokines that directly or indirectly modulate neuronal NLRP1 inflammasome activity. Moreover, our findings suggest more severe oxidative stress in the supernatant after AQP4 knockdown, which also promotes neuronal pyroptosis. Other potential mechanisms may also include on how AQP4 in astrocytes regulates neuronal pyroptosis. AQP4 regulates extracellular calcium homeostasis, the dysregulation of which can cause neuronal pyroptosis (47, 48). In addition, glutamate transporter-1 (GLT-1) is the primary glutamate transport channel in the brain. After AQP4 knockout, the expression of GLT-1 is significantly reduced (49). Therefore, AQP4 is closely related to glutamate transport. Dysfunction of AQP4 can lead to excessive accumulation of glutamate in the extracellular space, triggering excitatory neurotoxicity and resulting in neuronal pyroptosis (50).

Several limitations warrant consideration. Firstly, although AQP4 is predominantly expressed in astrocytes, our study focuses on neuronal pyroptosis. While we used a co-culture system to partially address neuron–astrocyte crosstalk, detailed astrocyte-specific mechanisms remain unexplored. The extracellular ionic homeostasis, glutamate metabolism, and signaling molecules should be intensively explored. Single-cell transcriptomics can be added to better study the interactions between neurons and astrocytes and uncover new mechanisms. Also, it may not be insufficient to establish that AQP4 inhibits neuronal pyroptosis solely through the NLRP1 inflammasome in the current study. Further verification would require manipulating the expression of other inflammasomes and observing corresponding changes in neuronal pyroptosis, which will be explored in subsequent research. Meanwhile, whether oxidative stress can be regarded as the upstream pathway of NLRP1 should be further validated using the related activators and inhibitors. Besides, the cellular sources of cytokines were not performed by cellular profiling and cytokine analysis via flow cytometry. The future work should employ flow cytometry coupled with cell-type-specific markers to precisely identify which cell populations contribute to the release of these inflammatory cytokines following ischemic injury. In addition, translation of these findings to humans requires caution, as AQP4 polymorphisms and distribution patterns vary among species.

## 5 Conclusion

In summary, this study demonstrates that AQP4 serves as a crucial endogenous protector against neuronal pyroptosis following ischemic stroke. Its deficiency exacerbates neurological injury, oxidative stress, and inflammatory responses, not only in the ischemic core but also in the remote contralateral cortex, implicating AQP4 in mitigating diaschisis. Mechanistically, we identify that AQP4 exerts its anti-pyroptotic effect by activating PI3K/Akt pathway as well as up-regulating IκBα, thereby inhibiting the canonical NF-κB signaling pathway and subsequent activation of the NLRP1/caspase-1/GSDMD axis. These findings delineate a novel AQP4/IκBα/NF-κB regulatory circuit in post-ischemic neuronal death. Targeting this pathway, either by enhancing AQP4 function or modulating its downstream signals, presents a promising dual-target therapeutic avenue for conferring both focal and global neuroprotection, potentially improving functional recovery after stroke. Future research should focus on astrocyte-specific mechanisms and the translational potential of these findings in human pathophysiology.

## Data Availability

The data presented in the study are deposited in the Zenodo repository, accession number doi: 10.5281/zenodo.18770747 with the link https://zenodo.org/records/18770748.
