# Supplementary material for: Aquaporin-4 suppresses neuronal pyroptosis after ischemic stroke via the IκBα/NF-κB signaling pathway
Source: Front Immunol. 2026 Mar 6;17:1778802. doi: 10.3389/fimmu.2026.1778802 (PMC13002381; doi:10.3389/fimmu.2026.1778802)
Supplement: Supplementary file 1 [file DataSheet1.pdf]

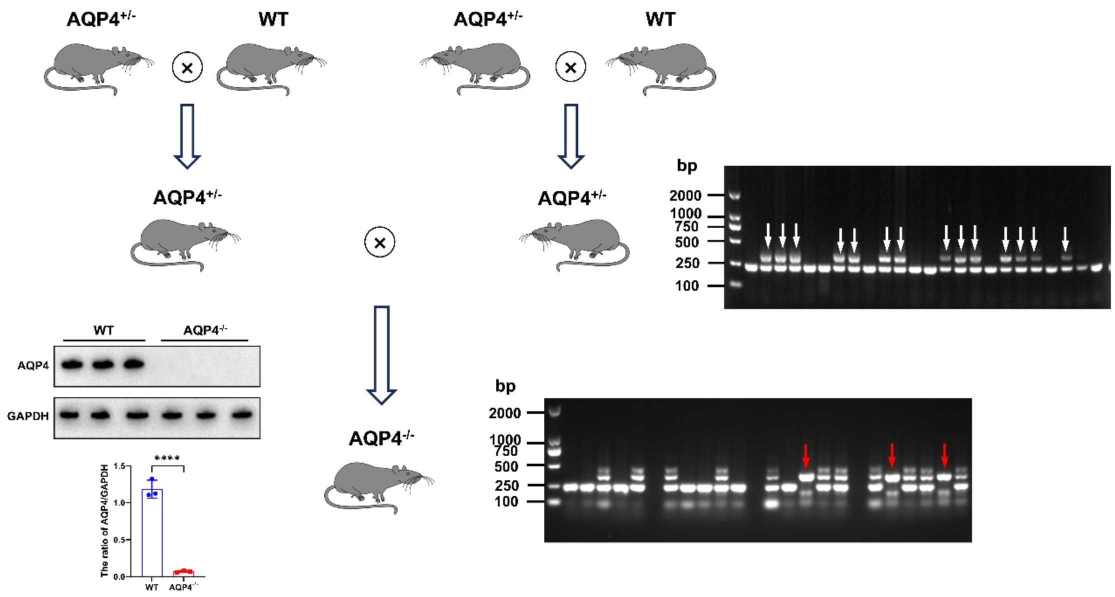


**Supplementary Figure S1. Generation of AQP4$^{-/-}$ mice.** In the representative images of PCR genotyping analysis, WT allele yielded a 240-base pairs (bp) product, heterozygote allele yielded a 240-bp and 320-bp product (white arrows), and hemozygote allele yielded a 320-bp product (red arrows). Western blot analysis confirming the absence of AQP4 protein expression in the brain tissues of AQP4$^{-/-}$ mice compared to WT mice. **** *P*<0.0001.
